# *Pseudomonas aeruginosa* RTE4: A Tea Rhizobacterium With Potential for Plant Growth Promotion and Biosurfactant Production

**DOI:** 10.3389/fbioe.2020.00861

**Published:** 2020-07-29

**Authors:** Ankita Chopra, Shishir Bobate, Praveen Rahi, Arun Banpurkar, Pranab Behari Mazumder, Surekha Satpute

**Affiliations:** ^1^Department of Biotechnology, Assam University, Silchar, India; ^2^Department of Microbiology, Savitribai Phule Pune University, Pune, India; ^3^National Centre for Microbial Resource, National Centre for Cell Science, Pune, India; ^4^Department of Physics, Savitribai Phule Pune University, Pune, India

**Keywords:** biosurfactant, *Pseudomonas*, PGPR, tea, surface tension

## Abstract

Tea is an ancient non-alcoholic beverage plantation crop cultivated in the most part of Assam, India. Being a long-term monoculture, tea plants are prone to both biotic and abiotic stresses, and requires massive amounts of chemicals as fertilizers and pesticides to achieve worthy crop productivity. The rhizosphere bacteria with the abilities to produce phytohormone, secreting hydrolytic enzyme, biofilm formation, bio-control activity provides induced systemic resistance to plants against pathogens. Thus, plant growth promoting (PGP) rhizobacteria represents as an alternative candidate to chemical inputs for agriculture sector. Further, deciphering the secondary metabolites, including biosurfactant (BS) allow developing a better understanding of rhizobacterial strains. The acidic nature of tea rhizosphere is predominated by *Bacillus* followed by *Pseudomonas* that enhances crop biomass and yield through accelerating uptake of nutrients. In the present study, a strain *Pseudomonas aeruginosa* RTE4 isolated from tea rhizosphere soil collected from Rosekandy Tea Garden, Cachar, Assam was evaluated for various plant-growth promoting attributes. The strain RTE4 produces indole acetic acid (74.54 μg/ml), hydrolytic enzymes, and solubilize tri-calcium phosphate (46 μg/ml). Bio-control activity of RTE4 was recorded against two foliar fungal pathogens of tea (*Corticium invisium* and *Fusarium solani*) and a bacterial plant pathogen (*Xanthomonas campestris*). The strain RTE4 was positive for BS production in the preliminary screening. Detailed analytical characterization through TLC, FTIR, NMR, and LCMS techniques revealed that the strain RTE4 grown in M9 medium with glucose (2% w/v) produce di-rhamnolipid BS. This BS reduced surface tension of phosphate buffer saline from 71 to 31 mN/m with a critical micelle concentration of 80 mg/L. Purified BS of RTE4 showed minimum inhibitory concentration of 5, 10, and 20 mg/ml against *X. campestris*, *F. solani* and *C. invisium*, respectively. Capability of RTE4 BS to be employed as a biofungicide as compared to Carbendazim – commercially available fungicide is also tested. The strain RTE4 exhibits multiple PGP attributes along with production of di-rhamnolipid BS. This gives a possibility to produce di-rhamnolipid BS from RTE4 in large scale and explore its applications in fields as a biological alternative to chemical fertilizer.

## Introduction

In India, Assam is one of the largest producers of tea with high export rate of around 53.60 million kg (April–June, 2019) which values nearly 174 million USD (Tea Board of India, Major country wise exports data^1^).

Tea plants are largely affected with foliar fungal diseases and also some bacterial disease (Plant protection code December 2018, Ver. 10.0 by Tea board of India^2^). To combat these biotic stress, various chemicals including pesticides are routinely used, which cause serious damage to the plantation soils (Chakraborty et al., 2013). Thus, exploring the potential of native microbes and their secondary metabolites is of utmost importance for these can later replace the harsh chemical fertilizers.

Over the years, there has been depletion in availability of agricultural lands. Additionally, crops in the available lands are exposed to large number of biotic and abiotic stress. In order to manage crop and fight crop diseases pesticides/fertilizers are routinely used. These chemical-based “Protective-Agents” in turn cause huge amount of environmental pollution thereby multiplying challenges in the field of agriculture. Exploring the potential of rhizosphere-associated bacteria as Plant Growth Promoting Rhizobacteria (PGPR) have been worked upon since many years (Adesemoye et al., 2009). PGPR benefits host plants by synthesizing phytohormone, solubilizing minerals in soil, fixing molecular nitrogen, controlling phytopathogens, producing antibiotics for disease suppression and others which has been reviewed by researchers (Ahemad and Kibret, 2014).

Along with their PGP attributes, exploring the potential of bacterial secondary metabolites proves to be beneficial (Singh et al., 2019). One such metabolite is biosurfactant (BS), a natural surfactant derived from microbes/plant origin, referred to as “Green Surfactant.” BS are reported to improve soil quality, involve in PGP activity, and degrade/solubilize pesticides. These green surfactants are also utilized as carbon source by existing soil microbes (Sachdev and Cameotra, 2013). Moreover, because of high biodegradability, stability during harsh conditions, low toxicity and production from renewable sources; these surfactants can be used widely in various industries such as agriculture, food, pharmaceutical, oil and petroleum (Deepika et al., 2016). BSs are divided into five major classes based on chemical structures viz: glycolipids, phospholipids, lipoproteins, polymeric and particulate surfactants (Desai and Banat, 1997). Most extensively studied BS is glycolipids. Rhamnolipid is a type of glycolipid mostly produced by *Pseudomonas aeruginosa* (Hassan et al., 2016).

*Pseudomonas* is ubiquitous gram negative, rod shaped motile bacteria largely found in the rhizosphere. They have been documented since many years as plant growth promoters, bioremediation agents and biocontrol agents. Several strains of *P. aeruginosa* are involved in plant growth promotion (Islam et al., 2018; Uzair et al., 2018) while some strains are known to be opportunistic pathogens (Lyczak et al., 2000). However, Jan et al. (2011) have reviewed that *Pseudomonas* isolated from close association of plants are beneficial and they exert antagonistic effect only against plant pathogens. Rhamnolipid type BS derived from *P. aeruginosa* can be employed as bioremediation agent (Rashedi et al., 2005) and even BS produced from *Pseudomonas* assist in uptake of hydrocarbons. *Pseudomonas* sp. has been reported to have strong communication with their host plant wherein they display numerous plant growth promoting (PGP) attributes (Santoyo et al., 2012) and represents as one of the most important in conferring biocontrol against pathogenic microbes. Therefore, exploiting traits of PGPR additionally having BS producing ability makes it an efficient player in agriculture fields. Recently, PGP *Pseudomonas* sp. producing BS was isolated from pesticide contaminated field (Hassen et al., 2018) and from the roots of common reed having hydrocarbon degrading potential (Wu et al., 2018). Thus, from literature it appears that *Pseudomonas* strains are one of the promising microbial systems which can be exploited for production of surface active agents. Studies have been conducted on BS produced by marine bacteria (Dhasayan et al., 2015; Hamza et al., 2017; Tripathi et al., 2018), bacteria from oil contaminated soil (Ebadi et al., 2018; Soltanighias et al., 2019), heavy metal contaminated soil (Sheng et al., 2008), rhizospheric bacteria of black pepper (Tran et al., 2008) and from variety of crop plants (Goswami and Deka, 2019). After an in-depth literature survey, we came to a conclusion that there is no single report on BS producing-PGP tea rhizobacteria. This encouraged us to explore the BS producing abilities of rhizobacterium *Pseudomonas aeruginosa* RTE4 isolated from tea rhizosphere of Rosekandy Tea garden located in Cachar district of Assam. In the current research work, we are assessing traits like PGP, antimicrobial, biofungicide and detailed physico-chemical characterization of BS produced by RTE4 is incorporated.

## Materials and Methods

### Isolation of Rhizobacterium RTE4

Soil samples were collected from a depth of 5–20 cm from Tea rhizosphere of Rosekandy Tea estate located at 24.69°N, 92.71°E situated in Cachar district of Assam, India. Soil samples collection was carried out during July 2018 (early monsoon) where considerable humidity encourages growth of various microorganisms in tea rhizosphere (see text footnote 2). The soil samples collected in sterile polythene bags were carried to the laboratory and stored at 4°C for further use. Isolation of bacterium RTE4 was carried out on Luria Bertani (LB) agar at 30°C for 24 h (Chopra et al., 2020).

### Identification of Rhizobacterium RTE4

Preliminary Characterization of RTE4 was carried out through colony morphology. Genomic DNA extraction was carried out using FavorPrep^TM^ Blood Genomic DNA Extraction Mini kit (FAVORGEN- Europe). The 16S rRNA gene was amplified using universal primers (27F:5′-AGAGTTTGATCCTGGCTCAG-3′ and 1492R:5′TACGGCTACCTTGTTACGACTT-3 ′) according to the methods described by Gulati et al. (2008), and the amplified product was directly sequenced using the ABI PRISM Big Dye Terminator v3.1 Cycle Sequencing kit on a 3730xl Genetic Analyzer (Applied BioSystems). Further, phylogenetic analysis was carried out using CLUSTAL X2 (Thompson et al., 1997). The best fit model test was carried out and phylogenetic tree was constructed using Neighbor-Joining (NJ) tree. Bootstrap analysis with 1,000 replicates was performed using MEGA7 software (Kumar et al., 2016). The culture RTE4 was deposited at National Centre for Microbial Resource (NCMR), Pune, Maharashtra, India under the general deposit with Accession no MCC 3945. The 16S rRNA gene sequence obtained was submitted to NCBI GenBank Data base with accession number-MK530435.

### Assessing RTE4 for Plant Growth Promoting and Antifungal Traits

Quantitative analysis of RTE4 for PGP traits such as indole acetic acid (IAA) production and phosphate solubilization was carried out as described by Mohite (2013) and Gulati et al. (2008) respectively.

Antifungal attributes included screening for production of hydrolytic enzymes viz. protease, cellulase and chitinase. Proteolytic activity was confirmed by halo formation around the spot of RTE4 inoculation on skim milk agar (SMA) plate incubated for 2–4 days at 28°C (Chu, 2007). Cellulase activity was screened by spot inoculating RTE4 on Cellulose Congo Red agar plates at 28°C for 72 h. Discoloration of Congo Red around the culture was an indicative of cellulase activity by the bacterium used in the screening test (Gupta et al., 2012). Chitinase production was qualitatively assessed by streaking a bacterial colony on 1% (w/v) colloidal chitin agar plate and incubated at 30°C for up to 8 days. Chitinase activity of the rhizobacterium was confirmed by the presence of a zone of clearance around the bacterial colony (Kuddus and Ahmad, 2013).

Antagonistic activity of RTE4 was screened against two Tea foliar fungal pathogens namely *Corticium invisium* MCC 1841 and *Fusarium solani* MCC 1842. Fungal mycelia plug was kept at the center of Potato Dextrose (PD) agar plates equidistant to which bacterial culture was inoculated. Plates were incubated at 28°C and observed upto168 h. Antagonistic activity of RTE4 was calculated on control and test plate for computing Growth Inhibition (GI%) (El-Sayed et al., 2014).

### Screening RTE4 for Antibacterial Traits

Antibacterial activity was checked by co-culturing plant pathogen *Xanthomonas campestris* (NCIM 5028) with RTE4 on Muller Hinton Agar (MHA) plates incubated at 30°C up to 48 h. Inhibition in growth of *X. campestris* was indicative of antibacterial property of RTE4.

### Screening RTE4 for Biosurfactant Production

BS production was checked by growing RTE4 culture in M9 minimal media supplemented with 2% (w/v) of five different carbon sources namely peptone, maltose, glucose, fructose and sucrose. Further BS production was assessed by conducting assays such as Drop collapse (DC), Oil displacement, Surface tension (SFT) measurement as described by Satpute et al. (2008). In DC assay, 25 μl drop of cell free supernatant (CFS) (from 12 to 120 h at 12 h interval) was placed on parafilm coated solid surface and observed for drop collapse. The drop spread or collapse is an indicative for presence of surfactant in sample. Oil displacement assay was carried out by adding 20 ml water in petriplate and adding 20 μl crude oil on water surface. 10 μl of CFS was then added on to the oil surface. Appropriate positive and negative controls were also included in the study. In presence of BS, oil gets displaced indicating a positive test. SFT measurements were taken on Optical Contact Angle Goniometer (OCA 15 Plus, DataPhysics Instruments GmbH, Germany).

### Biosurfactant Production and Purification

BS production was carried out in M9 minimal medium supplemented with 2% (w/v) glucose as sole carbon source. A seed culture (25 ml) of RTE4 was prepared by transferring a single colony from LB agar. After 24 h of incubation, 5% (v/v) of RTE4 seed culture was transferred as inoculum in a fresh fermentation media (150 mL) taken in 1 L Erlenmeyer flask. The complete production of BS was carried out at 30°C, 150 rpm for 7 days which was observed at 12 h intervals. Simultaneously biomass (g/L) during BS fermentation was determined by measuring dried cell weight (Waghmode et al., 2019). Additionally, different parameters like drop size (mm), SFT (mN/m), and optical density (at 600 nm) were checked at 12 h interval. All experiments were carried out in triplicates. The mean values were plotted along with standard deviation (SD) using software – GraphPad Prism 6.0 (GraphPad, La Jolla, CA, United States). After completing the fermentation process, BS was extracted from CFS by centrifuging culture broth at 8,000 *g* for 20 min at 4°C. This CFS was then subjected to acid precipitation by using 5 N HCl. The solvent system used for extraction was chloroform: methanol (2:1 v/v) at room temperature. Further to obtain BS, the organic phase was subjected to rotary evaporation (40°C). The viscous dark brown colored BS was further purified by Silica gel (100–200 mesh, Merck) column chromatography and eluted with gradient of chloroform: methanol ranging from100:0, 90:10, 80:20, …10:90, 0:100 (v/v). BS was successfully obtained in the fraction of a gradient 70:30 (chloroform: methanol). All the fractions thus collected were analyzed using thin layer chromatography (TLC) (Satpute et al., 2010) and compared with commercial rhamnolipid BS (AGAE Technologies, United States).

### Physico-Chemical Properties of Biosurfactant

#### Surface Tension, Critical Micelle Concentration and Interfacial Tension Measurement of Biosurfactant

Different concentrations (1 mg/L to 10 g/L) of purified BS product were prepared in phosphate buffer saline (PBS) to determine the Critical Micelle Concentration (CMC). SFT reduction of PBS by RTE4 BS was determined by pendant drop technique using Optical contact angle Goniometer (OCA 15 Plus, DataPhysics Instruments GmbH, Germany). CMC value was determined by plotting SFT versus BS concentration on semi-log scale. The interfacial tension (IFT) of purified RTE4 BS was measured at CMC value against *n*-hexadecane (HiMedia, India).

#### Contact Angle Measurement and Emulsification Activity of Biosurfactant

Four different surfaces were used for determining the Contact Angle (CA) of purified BS viz. glass slide (highly hydrophilic), overhead projector (OHP) sheet (intermediate hydrophilic-hydrophobic), Teflon (polytetrafluoroethylene-PTFE) tape and parafilm (highly hydrophobic) and measured by sessile drop method (Hamza et al., 2017). A 7 μl drop was placed on those different surfaces and CA was analyzed by Optical Contact Angle Goniometer (OCA 15+, DataPhysics Instruments, GmbH, Germany) using SCA 20 software. To determine emulsification properties; various oils like sunflower, sesame and coconut (local market) were added to CMC solution of the RTE4 BS (1:1 v/v), vortexed for 2 min and allowed to stand for 24 h (Płaza et al., 2014).

#### Stability Studies of Biosurfactant

Stability of the RTE4 BS was assessed at different pH and temperatures. CMC solution of the BS was prepared in different buffer solutions with pH ranging from 2 to 12 and incubated for 24 h followed by SFT measurement to check the effect of pH (Waghmode et al., 2019) on SFT reducing abilities of BS. CMC solution of BS was incubated for 24 h at different temperatures viz. −20, 4, 30, 37, 50, and 121°C (autoclave) and SFT was measured.

#### Determination of Ionic Character of Biosurfactant

For determination of ionic charge on the RTE4 BS, agar double diffusion method was used. In this method, two wells were made on soft agar (1% w/v) plate. One was filled with the RTE4 BS dissolved in phosphate buffer (pH 7) and the other with 20 mM synthetic surfactant solutions viz., SDS (anionic), CTAB (cationic), Aerosol OTLR and Tween 80 (non-ionic) (Rufino et al., 2014).

#### Analysis of Biosurfactant by Thin Layer Chromatography

Detection of glycolipids, sugars, amino acids and lipids was conducted on heat-activated TLC aluminum plates pre-coated with silica gel (Silica gel 60 F_254_, Merck KGaA, Darmstadt, Germany). For the detection, 90% pure rhamnolipid (AGAE Technologies, United States) was used as a reference. The samples were loaded and spots were allowed to dry completely. The plates were then placed in four different solvent systems (1) chloroform: methanol: glacial acetic acid (65:25:2), (2) butanol: ethanol: water (5:3:2), (3) hexane: ethyl ether: formic acid (80:20:2) and (4) butanol: acetic acid: water (12:3:5) and developed by different developing reagents namely anisaldehyde, diphenylamine, iodine and ninhydrin reagents for the detection of glycolipids, sugars, lipids and amino acids, respectively (Satpute et al., 2010).

#### Fourier-Transform Infra-Red Spectroscopy

RTE4 BS was chemically characterized by performing FTIR spectroscopy (PerkinElmer, United Kingdom). Analysis was done in mid-IR region with 20 scan speed (Das et al., 2008).

#### Nuclear Magnetic Resonance Spectroscopy

For structure prediction, ^1^H and ^13^C proton nuclear magnetic spectra were recorded at 294 K on a 500 and 101 MHz nuclear magnetic resonance (NMR) spectrophotometer (Bruker, Germany). The samples were prepared as solutions in 100% CDCl_3_, with 5 mg of RTE4 BS and tetramethylsilane (TMS) as internal standard (Pemmaraju et al., 2012).

#### Liquid Chromatography-Mass Spectrometry (LC-MS/MS)

Electrospray ionisation mass spectra (ESI-MS) of RTE4 BS were recorded on 6550 UHD. Accurate Mass QTOF MS (Agilent Technologies, Santa Clara, CA, United States) was determined for RTE4 BS. Stock solution of compound was prepared by dissolving 2 mg substance in 1 ml chloroform: methanol (1:1 v/v). Aliquots of 0.1 ml were diluted in a mixture of acetonitrile – water (40:60, v/v) and introduced into mass spectrometer HPLC equipped with an Eclipse XDB C18 (150 mm × 4.6 mm, 5 m) reverse phase column with HPLC flow rate of 0.25 ml/min. ESI mode was as follows: negative mode [M-H]- at capillary and cone voltage of 2.65 kV and 40 V respectively with temperature at 250°C. The scanning mass range was from 150 to 800 Da. Argon was used as collision gas at collision –induced dissociation (Lotfabad et al., 2010).

### Determination of Antimicrobial Activities of Biosurfactant Produced by RTE4

Antimicrobial activity of BS from RTE4 was tested against Tea pathogenic fungi (two) and bacterium (one) namely *C. invisium*, *F. solani* and *X. campestris* respectively. For antifungal activity, a 6 mm fungal disc was placed at the center of PD agar plates surrounded by wells containing different concentrations of RTE4 BS at 5, 10, 15, and 20 mg/ml. Standard rhamnolipid was used as positive control and sterile PBS was used as negative control. *C. invisium* containing plates were then incubated at 28°C for 4 days while *F. solani* plates were incubated for 7 days. Antagonistic activity observing mycelia growth inhibition in test plate when compared to control plate covered with fungi. Antibacterial activity was screened on MHA plate having a lawn of *X. campestris* with 10^8^ CFU/ml. The wells on MHA plate were filled with 0.1 ml RTE4 BS of varying concentrations (as mentioned above) incubated at 28°C for 24 h to observe the zone of inhibition caused by RTE4 BS.

### Microdilution Assay to Determine Minimum Inhibitory Concentration of Biosurfactant

To assess minimum inhibitory concentration (MIC) of RTE4 BS against *C. invisium* and *F. solani* microdilution method suggested by Elshikh et al. (2016) was performed with slight modification. Briefly 20 μl BS solution of different concentrations ranging from 5, 10, 15 up to 50 mg/ml were added into wells containing 120 μl of PD broth. In control wells, 20 μl of PBS was added instead of BS. 10 μl of homogenous fungal suspension was added to each well. In three wells of both test plates no fungal suspension was added to check the sterility of the medium. Commercially available fungicide Carbendazim (Sigma Aldrich) of similar concentrations and BS (1:1) were added to the other wells containing fungal suspensions. The plates were incubated at 4°C for 1 h followed by incubation at 28°C for 5 days. The experiment was carried out in triplicate and fungal growth in each well was observed through naked eyes under bright light. BS treated wells were screened and compared with control wells having dense mycelia growth.

## Results

### Identification of RTE4

**FIGURE 1:**
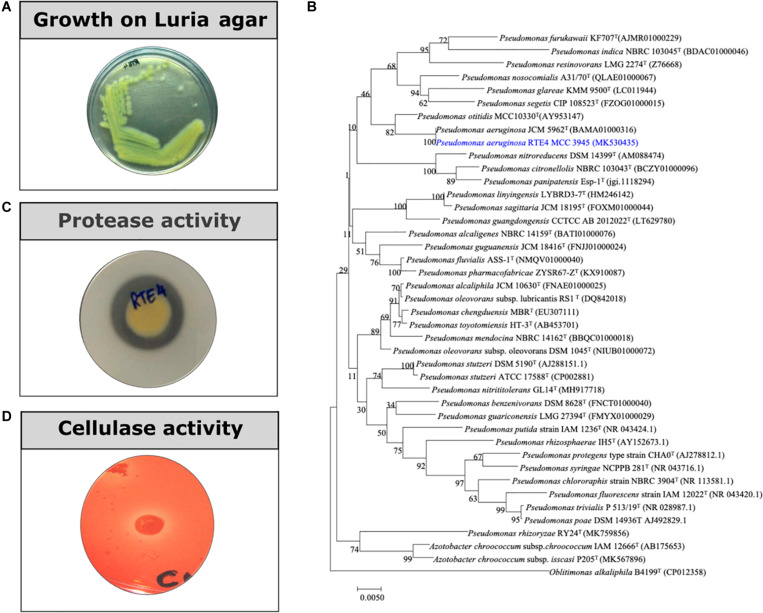
**(A)** A 24 h old culture of *Pseudomonas aeruginosa* RTE4 grown on Luria agar. **(B)** Phylogenetic tree based on the 16S rRNA gene sequences, showing the relationship between the strain RTE4 and other members of the genus *Pseudomonas*. The tree was reconstructed by the neighbor-joining method using the Kimura 2-parameter method (rooted with *Oblitimonas alkaliphile* B4199^T^ as an outgroup). Bootstrap values (expressed as percentages of 1,000 replications) above 50% are shown at branch points. Bar, 0.005 substitutions per nucleotide position. **(C)** Protease activity of RTE4 on skim milk agar plate. **(D)** Cellulase activity of RTE4 on cellulose Congo red agar plate. Clear halo around spot inoculated RTE4 is an indicative of cellulose production by RTE4.

Rhizobacterial strain RTE4 (Figure 1A) isolated from Rosekandy Tea garden situated in Cachar, Assam, India was identified as *Pseudomonas aeruginosa* RTE4 (GenBank Accession Number-MK530435). The identification of RTE4 using 16S rRNA gene sequencing revealed it as a phylogenetic neighbor of *Pseudomonas aeruginosa* (Figure 1B) with 99% identity. The amplification of 16S rRNA gene generated a single band of 1,380 bp for the strain RTE4.

### Plant Growth Promoting, Antifungal and Antibacterial Traits of RTE4

IAA production was quantified for 7 days old CFS of RTE4 grown in tryptophan supplemented Yeast Mannitol Broth. Supernatant on reacting with Salkowski reagent showed up to 74.54 μg/ml IAA production. For phosphate solubilization, in NBRIP broth media till 6 days, RTE4 solubilized in tri-calcium phosphate up to 47.83 μg/ml.

**FIGURE 2:**
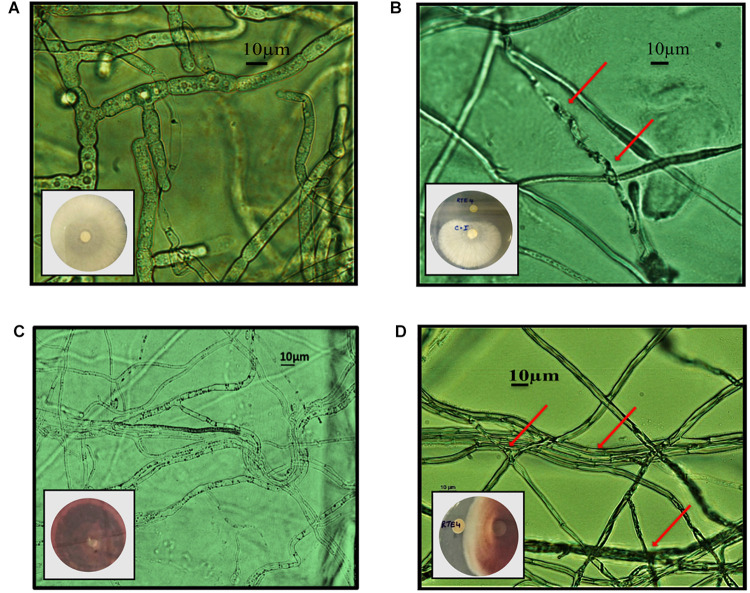
*In vitro* interaction of *Pseudomonas aeruginosa* RTE4 against two Tea fungal pathogens grown on potato dextrose agar and their morphological alterations under microscope (10×) panel **(A)** is the microscopic image of *Corticium invisium* (Control) without any distortion and panel **(B)** shows interaction of RTE4 with *C. invisium* having distorted mycelia (Test) panel **(C)** is the microscopic image of *Fusarium solani* (Control) without any distortion and panel **(D)** shows interaction of RTE4 with *F. solani* (Test) having narrowed mycelia and lack of pigmentation. The plate images are shown as insets in the respective microscopic images.

**FIGURE 3:**
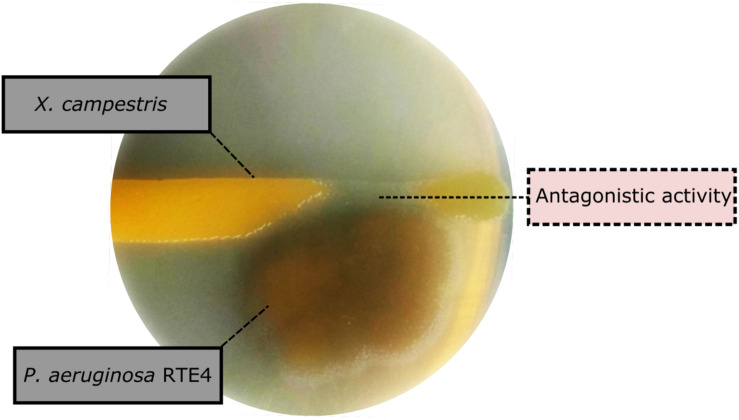
Biocontrol activity of *Pseudomonas aeruginosa* RTE4 (Light brown color) against *Xanthomonas campestris* (Yellow colored) after 2-day incubation at room temperature. Point of antagonism is shown by dotted lines.

Through *in vitro* assays, hydrolytic enzyme production by RTE4 showed secretion of protease and cellulase enzyme evident by formation of halos measuring around 8 mm on skim milk agar (Figure 1C) and 6 mm on cellulose agar plate respectively (Figure 1D). The isolate also produced chitinase. RTE4 also showed strong antagonistic activity against both Tea plant pathogens *C. invisium* and *F. solani* with GI percentage of 36.6 and 56.8 respectively. Microscopic images of *C. invisium* (Figure 2A) revealed distorted hyphae when cultured in presence of RTE4 (Figure 2B). *F. solani* (Figure 2C) also displayed narrow hyphae growth when grown in presence of RTE4 (Figure 2D). When checked against *X. campestris*, RTE4 showed promising antagonism in dual culture assay (Figure 3).

### Physical Characterization

#### Determination of Surfactant Activity

**FIGURE 4:**
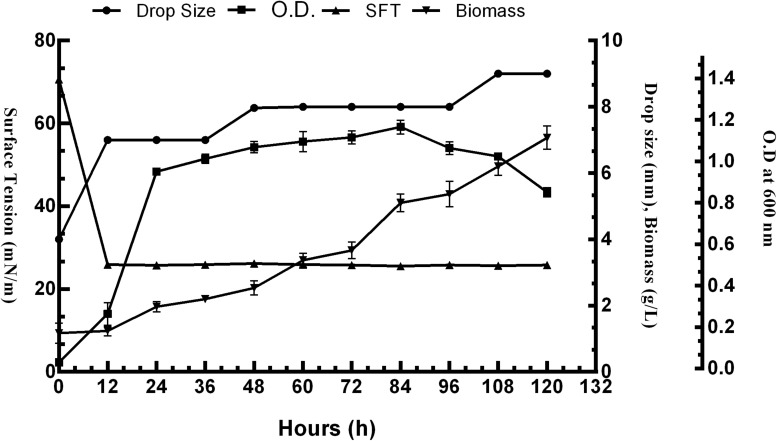
Growth and production of biosurfactant by *Pseudomonas aeruginosa* RTE4 in M9 minimal media with 2% (w/v) glucose as carbon source.

The surface activity of BS obtained from *P. aeruginosa* RTE4 (MCC 3945) was proved by several screening procedures viz. DC, OD, SFT reduction, IFT, CA and EI. All the results obtained were comparable with both positive (sodium dodecyl sulfate: SDS) and negative (water) controls. Out of all five different carbon sources, M9 media supplemented with 2% (w/v) glucose was found to be the most suitable for BS production by RTE4. A sharp decrease in SFT value of growth medium was observed only after 12 h of culture growth which continued to be the same till 120 h even when there was a gradual increase in the cell density. Increase in biomass of the organism was observed till 120 h. The CFS was capable of reducing the SFT from 70.62 to 31 mN/m. The pH of the culture broth was 7 and it remained unchanged throughout the experiment. The DC size of CFS increased from 4 to 8 mm in comparison with uninoculated medium with prolonged incubation of culture broth from 0 to 120 h (Figure 4).

**FIGURE 5:**
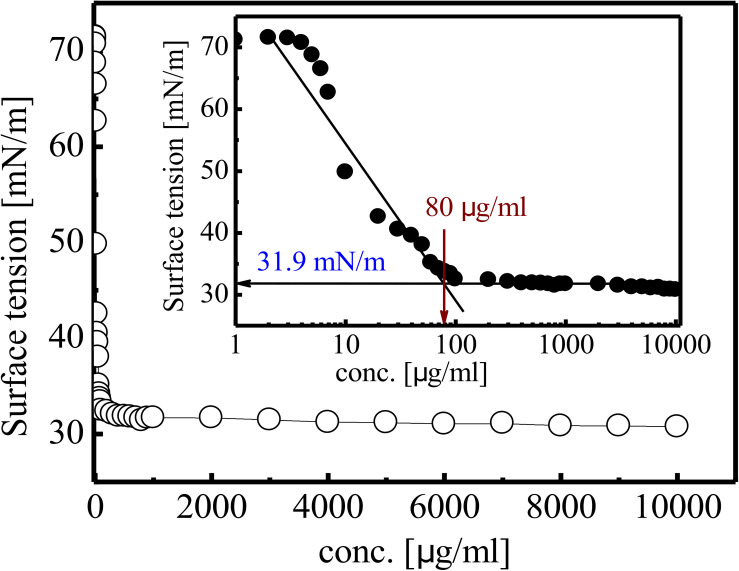
Variation in SFT of biosurfactant obtained from *Pseudomonas aeruginosa* RTE4. Inset showing exponential reflection of CMC value of SFT (mN/m) versus BS concentration (mg/L).

Gradual reduction in SFT of PBS (pH 7) was observed as inverse function of the BS concentration till CMC value. SFT of PBS was reduced from 71 to 31 mN/m at CMC value of 80 mg/L (Figure 5). The purified RTE4 BS reduced IFT of hexadecane interphase from 26.25 to 2.3 mN/m. IFT represents the force of attraction believed to be present between two liquids or fluids. Thus, the IFT value reported here between hexadecane-water interfaces certainly represents RTE4 BS as a promising candidate.

#### Contact Angle Measurement and Emulsification Activity

**FIGURE 6:**
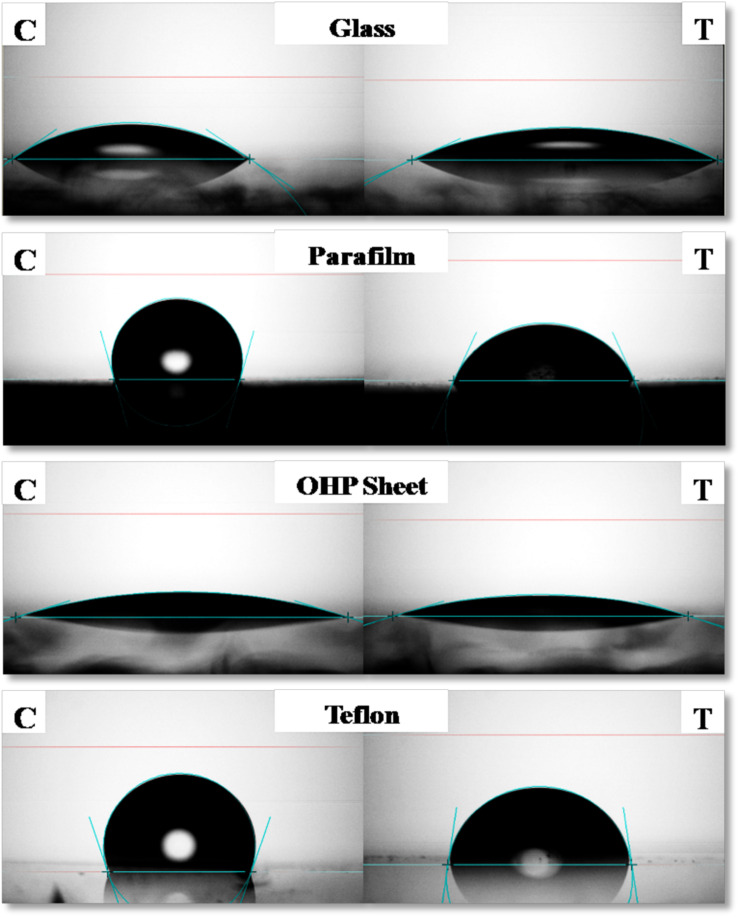
Measurement of contact angle (CA) of phosphate buffer saline (PBS) (Left side column indicating: Control) and CMC solution (80 μg/ml prepared in PBS) of biosurfactant obtained from *Pseudomonas aeruginosa* RTE4 (right side column indicating: test) on Glass (first row), Parafilm (second row), OHP sheet (third row), and Teflon (fourth row).

Contact Angle of RTE4 BS solution (at CMC) was measured on different surfaces by using PBS as a negative control. The maximum reduction in CA was observed on parafilm from θ = 105° to 66°;. On highly hydrophobic Teflon, CA was reduced from θ = 109° to 82°. Slight decrease of CA was observed on glass (θ = 35° to 25°) and OHP sheet (θ = 17.5° to 16.8°). This indicates ability of RTE4 BS to wet hydrophobic surfaces (Figure 6). There was lack of formation of stable emulsion in case of sesame oil and sunflower oil (E_24_ = 5.5 and 4%, respectively) while better emulsification was observed for coconut oil (E_24_ = 53.8%). Thus, RTE4 BS demonstrated good surface wetting properties (parafilm and Teflon) with poor emulsifier properties.

#### Stability Studies of Biosurfactant

**FIGURE 7:**
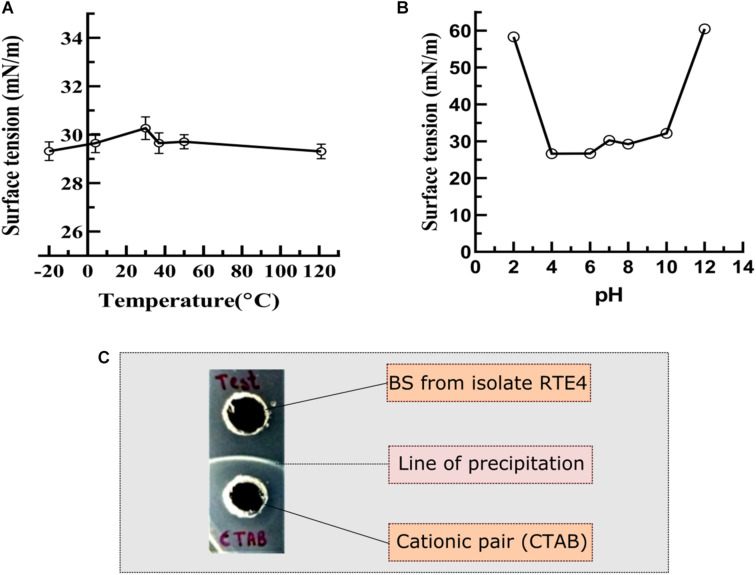
Measurement of surface tension to determine the stability of biosurfactant derived from *Pseudomonas aeruginosa* RTE4. **(A)** At different temperature. **(B)** At different pH. **(C)** Determination of ionic character for BS derived from *P. aeruginosa* RTE4 using agar plate double diffusion assay against Cetyltrimethyl ammonium bromide (CTAB-cationic nature). Black colored arrow indicates line of precipitation formed between anionic (BS from RTE4) cationic (CTAB) pair.

The RTE4 BS appeared to be stable at pH range 4–8 as indicated by SFT determination after 24 h. Extreme acidic and alkaline conditions significantly reduced SFT capacity of the BS (Figure 7B). The BS retained its surface acting properties after incubation at all the temperatures mentioned above (−20 to 121°C) and even after autoclaving (Figure 7A).

#### Ionic Character Determination

In the double diffusion method, compounds with opposite charges form a line of precipitation when they encounter each other. A line of precipitation was observed between CTAB (cationic) and the RTE4 BS (anionic) while there was lack of such line between SDS (anionic) and the RTE4 BS. This confirms the anionic nature of RTE4 BS (Figure 7C).

### Chemical Characterization

#### Analysis by Thin Layer Chromatography

**FIGURE 8:**
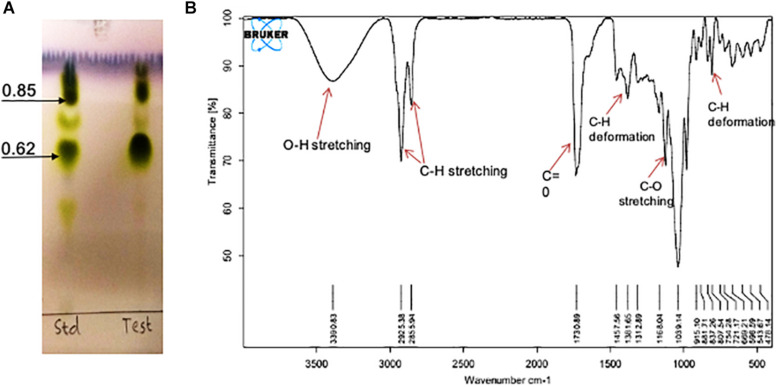
Chemical characterization of *Pseudomonas aeruginosa* RTE4 derived biosurfactant (Test) by using **(A)** Thin Layer Chromatography (TLC): TLC plate run in a solvent system of chloroform: methanol: water (65:25:4) and developed with anisaldehyde reagent (H_2_SO_4:_ anisaldehyde: glacial acetic acid; 1: 0.5: 50) and indicated typical green color spots of rhamnolipid **(B)** FTIR spectrum.

On TLC plate developed with anisaldehyde reagent, the purified RTE4 BS exhibited two typical green spots with *R*_*f*_ values 0.62 and 0.85, same as that of the reference rhamnolipid, indicating RTE4 BS to be a mixture of mono and di-rhamnolipid). TLC for lipid and sugar indicated presence of typical brown and green colored respectively (matching with rhamnose) proving the glycolipid type of BS (Figure 8A).

#### Fourier-Transform Infra-Red Spectroscopy

The IR spectra showed a broad peak at 3,390 cm^–1^ symmetric for hydroxyl group (-OH) implying presence of polysaccharides. The stretch showed sharp bands at 2,925 and 2,855 cm^–1^ indicating stretching vibrations of alkyl group (CH_2_- CH_3_) of hydrocarbon chain. Intense peak was observed at 1,730 cm^–1^ and weak peak at 1,457 cm^–1^ denoting presence of ester carbonyl groups (C=O in COOH). In our study we found peaks at 1,381, 1,312, 1,457 and 807 cm^–1^ denoting plane deformation of C-OH, O-H and C-H band respectively. However, C-O stretching was observed only at 1,168 cm^–1^ (Figure 8B). Results were compared with standard rhamnolipid sample as well (Supplementary Figure S1).

#### Nuclear Magnetic Resonance Spectroscopy

**FIGURE 9:**
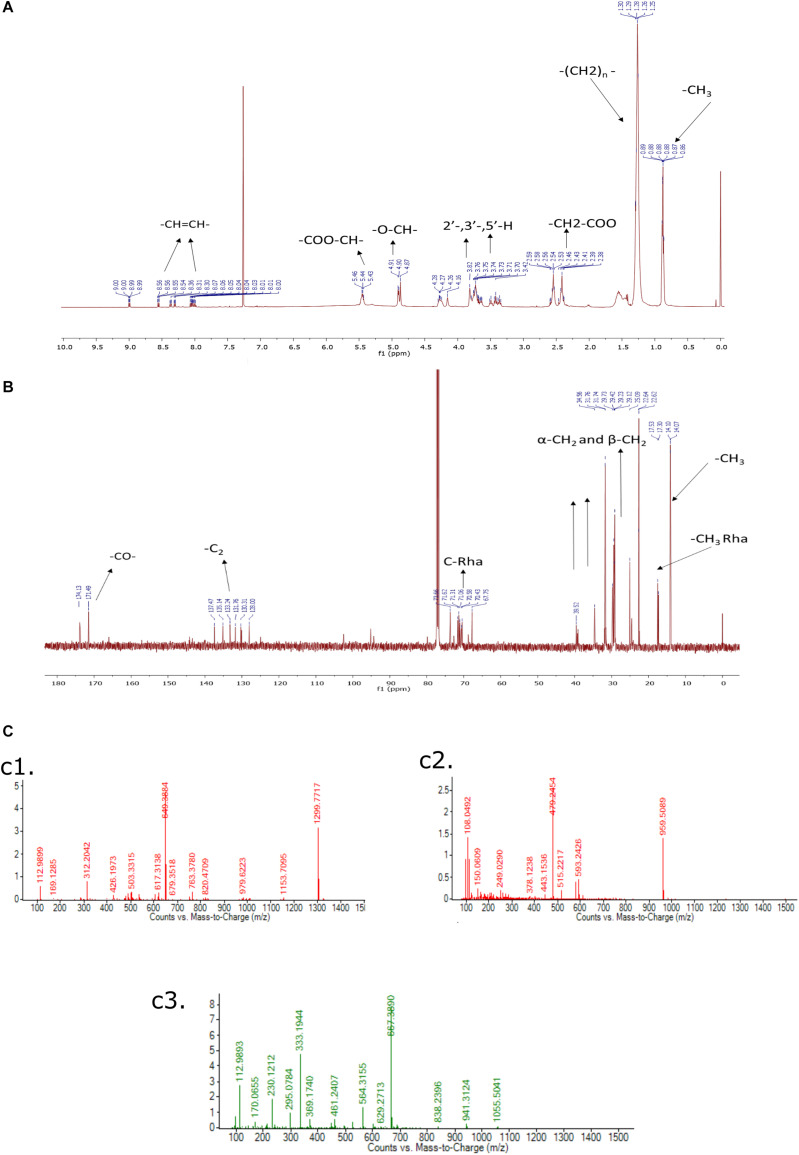
**(A)** ^1^H NMR (500 MHz, CDCl_3_) of Di-Rhamnolipid, **(B)** ^13^C NMR (126 MHz, CDCl_3_) of Di-Rhamnolipid. **(C)** LC-MS spectrum of biosurfactant produced by *Pseudomonas aeruginosa* RTE4. ESI-MS confirms the highest intensity di-rhamnolipid **(c1)**; palmityl palmitate **(c2)**; dipalmitin **(c3)**.

The purified rhamnolipids were confirmed by characteristic chemical shifts ^1^H and ^13^C NMR and the chemicals shift (ppm) values are depicted in Figures 9A,B and were compared with available literature (Lotfabad et al., 2010; Twigg et al., 2018). The obtained results^1^H NMR (500 MHz, CDCl_3_) δ 0.88 (-CH_3_), 1.28 (-(CH_2_)_n_), 2.4–2.68 (-CH_2_-COO-), 3.40–3.80 (2-, 3-,5-H), 4.7–4.8 (-O-CH-), 5.43–5.46 (-COO-CH-), 8.00–8.50 (-CH = CH-), and ^13^C NMR (126 MHz, CDCl_3_) δ14.07 (CH_3_), 17.53 (-CH_3_Rha), 20.0–40.0 (-CH_2_-), 65.0–75 (C-Rha), 128.0–141.0 (-CH = CH-), 171.0–174.0 (-CO-) confirms the di-rhamnolipids.

#### Liquid Chromatography-Mass Spectrometry (LC-MS/MS)

In this analysis, the highest intensity rhamnolipid was found the di-rhamnolipid homologs which corresponds to the deprotonated molecules of Rha2-C10-C10 (di-rhamnolipid) and detected as the anions of m/z = 649.38 (ca. 80%). Their fractions are daughter ion of m/z 493 exhibiting the rupture of the ester bond between the two alkylic chains in the di-rhamnolipid depicted in Figure 9C,c1 with traces of palmityl palmitate (m/z = 479.24, Figure 9C,c2 and dipalmitin (m/z = 667.38, Figure 9C,c3. This analysis results are compared with previous reports dealing with synthesis of rhamnolipid surfactant mixtures in which di-rhamnolipid was the major component (Behrens et al., 2016; Twigg et al., 2018) with traces of palmityl palmitate, dipalmitin, and Oleic acid. Standard rhamnolipid sample was also used to compare and confirm the presence of di-rhamnolipid in BS produced by RTE4 isolate (Supplementary Figure S2).

### *In vitro* Assessment of MIC for Fungal Pathogens by RTE4 Biosurfactant

**FIGURE 10:**
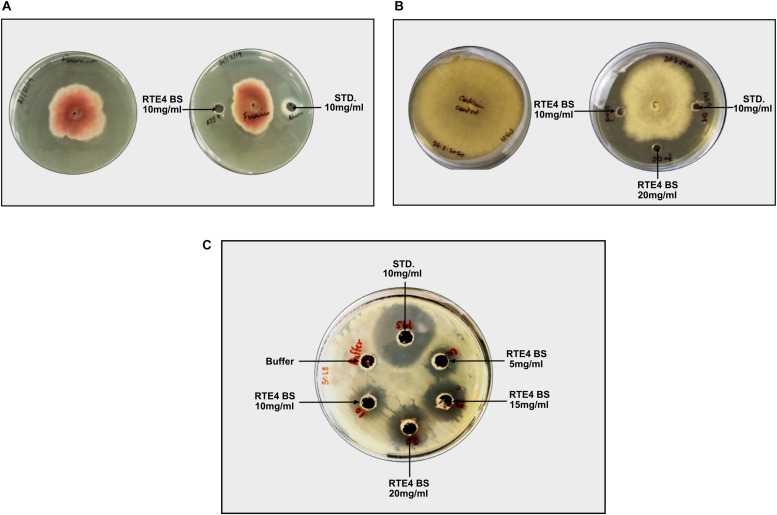
Biocontrol potential of *Pseudomonas aeruginosa* RTE4 derived BS against *Fusarium solani* **(A)** and *Corticium invisium* **(B)**. Well containing RTE4 BS (10 mg/ml) shows antagonism against *F. solani* as compared to control whereas RTE4 BS of 20 mg/ml concentration shows antagonism against *C. invisium* as compared to control. Arrow indicates point of antagonism. **(C)** Biocontrol potential of *P. aeruginosa* RTE4 derived BS against a lawn of *X. campestris* NCIM 5028. Wells containing different concentration (5, 10, 15, and 20 μg/ml) of RTE4 BS showing zone of inhibition on lawn of *X. campestris* grown on Muller Hinton agar plate with buffer as negative control. Commercial rhamnolipid was also used as reference compound (10 mg/ml).

Antagonistic activity for *C. invisium* was evident at MIC of 20 mg/ml concentration of RTE4 BS in both plate assay and microdilution method (Figure 10A). MIC for *F. solani* was found to be 10 mg/ml (Figure 10B). Carbendazim was found to inhibit fungal growth at initial concentration of 1 mg/ml. No fungal growth was observed in wells uninoculated (control) with fungal suspension. In control wells without addition of RTE4 BS or carbendazim heavy fungal growth was observed. Growth of *X. campestris* was inhibited at MIC of 5 mg/ml as observed in plate assay (Figure 10C).

## Discussion

Increased use of chemical fertilizers is a matter of great concern in agriculture. Application of native rhizobacteria to be employed as biofertlizers is momentously appreciated. For this, it is mandatory to understand the PGP potential of microbes dwelling in the rhizosphere. There are several metabolites secreted by bacteria which contribute toward growth and health of the host plant but remains unknown. Microbial BS are advantageous over chemical surfactants in various properties, both physical and chemical (Desai and Banat, 1997). The phenomenon of bacterial quorum sensing (QS) is mediated via acyl homoserine lactones (acyl-HSLs) signaling molecules and are also known to regulate PGPR traits. QS and acyl-HSLs may have a significant impact on the synthesis of BS in *Pseudomonas* sp. (Reis et al., 2011). Therefore, further exploring QS potential of the isolate will help us to enhance our knowledge on the role of QS in BS production in the isolate RTE4. Among the diverse bacterial species dwelling in Tea rhizosphere, *Pseudomonas* sp. is one of the dominantly present bacteria. In the present work, an isolate RTE4 identified as *P. aeruginosa* isolated from Tea-root adhered soil was assessed for its PGP traits, biocontrol potentials, BS production and its physical-chemical characterization. To evaluate PGP efficacy of RTE4 isolate, IAA and phosphate solubilization were quantified. Later biocontrol activity of RTE4 and BS from RTE4 was screened against foliar tea pathogenic fungi *C. invisium* and *F. solani* and bacteria *X. campestris*. BS is also reported to be one of the traits contributing to the antagonistic potentials (Caulier et al., 2018). Therefore, in current study we also investigated if BS from RTE4 confers biocontrol potential to the tea plant pathogens. Further investigation on other physical and chemical properties of RTE4 BS helps us in understanding its efficiency for commercial application.

The rhizobacterium RTE4 (MCC 3945) having typical morphological features like gram negative rod shaped, motile, catalase positive was identified successfully by phylogenetic analysis signature sequence as *Pseudomonas aeruginosa*. Among PGP traits, IAA production is considered to be one of the major traits (Spaepen et al., 2007). Apparently, IAA has no function in bacterial cells but it is hypothesized to improve plant-microbe interaction by increasing root colonizing efficiency, root development and nutrient uptake for plants (Etesami et al., 2015). RTE4 produces plant phytohormone IAA upto 74.54 μg/ml. Several nutrients are required by plant for its growth and development. Phosphate is second most important macronutrient required by plants after nitrogen. Unlike nitrogen, phosphate cannot be made biologically available from the atmosphere, hence lies the importance of phosphate solubilizing bacteria which help plants to solubilize inorganic phosphate present in soil (Rodrìguez and Fraga, 1999). When cultivated in National Botanical Research Institute’s phosphate growth medium (NBRIP) media at 28°C, RTE4 was able to solubilize inorganic tricalcium phosphate present in the medium from 27.77 μg/ml up to 47.83 μg/ml till sixth day.

Since many years, *Pseudomonas* sp. are known to be used as biocontrol agents (Islam et al., 2018). Along with other traits such as antibiotics, proteolytic and cellulolytic enzymes, volatile compounds, siderophores, BS is also one among them. Screening assays conducted for RTE4 demonstrated antifungal traits and as well as protease and cellulase activities. Strong antagonistic activity of RTE4 was observed against fungal pathogens *C. invisium* and *F. solani* and bacterial pathogen *X. campestris*.

The metabolic pathway involving glucose for microbial production of rhamnolipid is well characterized. Glucose is a preferable carbon source for it can be converted to precursor molecules required for rhamnolipid synthesis (Tan and Li, 2018). In our study also, it was found that among all other carbon sources tested, glucose proved to be the best one for the growth and production of BS from *P. aeruginosa* RTE4. A sharp decline in SFT of culture broth was recorded during logarithmic phase (12 h) of RTE4. The SFT reduced from 71 to 31 mN/m at 12 h and no further decline was observed upon incubation of culture up to 120 h meaning that RTE4 produces BS at logarithmic phase as a primary metabolite (Liu et al., 2018). However, pH of the media remained unchanged throughout incubation of RTE4. The SFT decreased significantly with increased concentration of RTE4 BS. CMC value can be used as a measure of quality of BS. Efficient BS have lower CMC and are required in less quantity to reduce the SFT of liquids. The CMC of RTE4 BS was determined to be 80 mg/L, suggesting surface activity at lower concentrations. Researchers have reported CMC range for *P*. *aeruginosa* is 5–200 mg/L (Hörmann et al., 2010; Müller et al., 2010) and SFT reduction from 72 to 30 mN/m (Varjani and Upasani, 2017). At CMC value, CA of water on hydrophobic substrates decreased significantly.

RTE4 BS sample was also investigated for thermal and pH stability by measuring the SFT of BS containing solution. It was found that the BS from RTE4 remained stable at acidic pH. This property could be attributed to the fact that bacteria in the tea rhizosphere may have acclimatized to the acidic soil conditions of tea garden (Borah et al., 2019). For commercial application of BS, its thermal stability is of utmost importance (Sharma et al., 2014). BS from RTE4 also maintained its stability at varying temperature conditions from −20 to 121°C making it efficient for commercial applications.

For extraction of BS, we found chloroform: methanol (2:1, v/v) as a suitable solvent system as previously reported (Cheng et al., 2017). Analysis of purified BS through analytical techniques like TLC, FTIR, NMR and LC-MS revealed a rhamnolipid nature. The stretching vibrations of different chemical groups in FTIR analysis is in agreement with previous studies reported presence of rhamnolipid-like BS from *P. aeruginosa* (Rikalovic et al., 2012; Khademolhosseini et al., 2019). Chemical shifts in ^1^H and ^13^C NMR spectra indicates purified product from *P. aeruginosa* RTE4 to be a di-rhamnolipid. This was in agreement with (Lotfabad et al., 2010). In addition to NMR analysis, the structural composition was identified through LC-MS illustrated the presence of peaks of molecular masses corresponding to the members of lipids and sugars moieties. Di-rhamnolipids were found to be the major component. TLC of purified RTE4 BS showed presence of mono and di-rhamnolipid. Thus, we confirm the BS produced by *Pseudomonas* RTE4 is of rhamnolipid type.

Rhamnolipid produced by *P. aeruginosa* is reported to confer biocontrol against *F. verticillioides* (Borah et al., 2016). No study has been conducted so far which explores the potential of native tea PGPR and its BS in conferring biocontrol against tea foliar pathogens used in this study. Pathogenic fungi and bacteria analyzed were susceptible to the rhamnolipid extract of RTE4 and to standard rhamnolipid as well. However, it is further essential to understand the mechanism by which BS portrays antagonism. We tried to compare the effect of commercially available fungicide, carbendazim, commonly used in agricultural field with that of BS from RTE4 in controlling fungal growth. It was observed that BS of RTE4 could control fungal growth to a considerable extent with increasing concentration of 15, 20 upto 50 mg/ml. Although acceptable daily intake (ADI) of carbendazim. India is 0.03 mg/kg/day (Sharma, 2007) yet repeated use of carbendazim is a matter of serious concern for health and safety of all the organisms (Singh et al., 2016). BSs are already applied as biopesticides in agricultural field (Sachdev and Cameotra, 2013). However, it is mandatory to explore more microbes for rhamnolipid production considering the huge demanding global BS market (Sekhon Randhawa and Rahman, 2014). We made an attempt to exploit the native bacteria of tea rhizosphere to understand the nature of BS it produces with its antimicrobial properties. To meet the fungicidal efficiency of BS in fields, large scale BS production strategy needs to be explored such that BSs can be used as biofungicide.

## Conclusion

Efficacy of a microbe to be exploited as biofertlizers first needs an understanding of the mechanisms by which it shows plant beneficial traits. In environment many bacteria which proves to be beneficial for plants are controversial for being pathogenic to humans. Therefore, digging out the targeted secondary metabolites from such plant- native microbes eliminates the fear of its virulence to humans. *P. aeruginosa* is one such controversial species which is widely found in various plant rhizosphere and yet claimed to be a PGP. In current study, we isolated a PGPR *P. aeruginosa* RTE4 from tea rhizosphere of Assam, India. Along with PGP traits such as IAA production, solubilization of inorganic phosphate, production of hydrolytic enzymes, it also shows strong antagonistic activity against two selected foliar pathogenic fungi of tea. We focused on understanding the type of BS it produces and if the BS thus produced can be made commercially available. Extensive physico-chemical characterization revealed BS of rhamnolipid type and demonstrated significant reduction in SFT, IFT and CA with low CMC value (80 mg/L) of RTE4 BS which proved its surfactant properties. Further, this compound possesses promising antimicrobial properties. Thus, finally we conclude that the strain RTE4 exhibits multiple PGPR attributes along with production of BS molecules having huge potential for their applications in agriculture as biofungicide.

## Data Availability Statement

All datasets presented in this study are included in the article/Supplementary Material.

## Author Contributions

AC, SB, and AB performed the experiments. PR, AB, PM, and SS conceived the idea. All authors designed the study, analyzed the results, and wrote the entire manuscript. All authors read and approved the final manuscript.

## Conflict of Interest

The authors declare that the research was conducted in the absence of any commercial or financial relationships that could be construed as a potential conflict of interest.

## Footnotes

1 http://www.teaboard.gov.in/TEABOARDPAGE/NTg

2 http://www.teaboard.gov.in/TEABOARDPAGE/ODY

## Supplementary Material

FIGURE S1 FTIR spectrum of commercial standard rhamnolipid.

FIGURE S2 LC-MS spectrum of commercial Standard rhamnolipid. ESI-MS confirms the highest intensity Di-Rhamnolipid **(A)**; Palmityl palmitate **(B)**; Dipalmitin **(C)**.
