# Supplementary figures and images for: Pseudomonas aeruginosa RTE4: A Tea Rhizobacterium With Potential for Plant Growth Promotion and Biosurfactant Production
Source: Front Bioeng Biotechnol. 2020 Jul 29;8:861. doi: 10.3389/fbioe.2020.00861 (PMC7403194; doi:10.3389/fbioe.2020.00861)

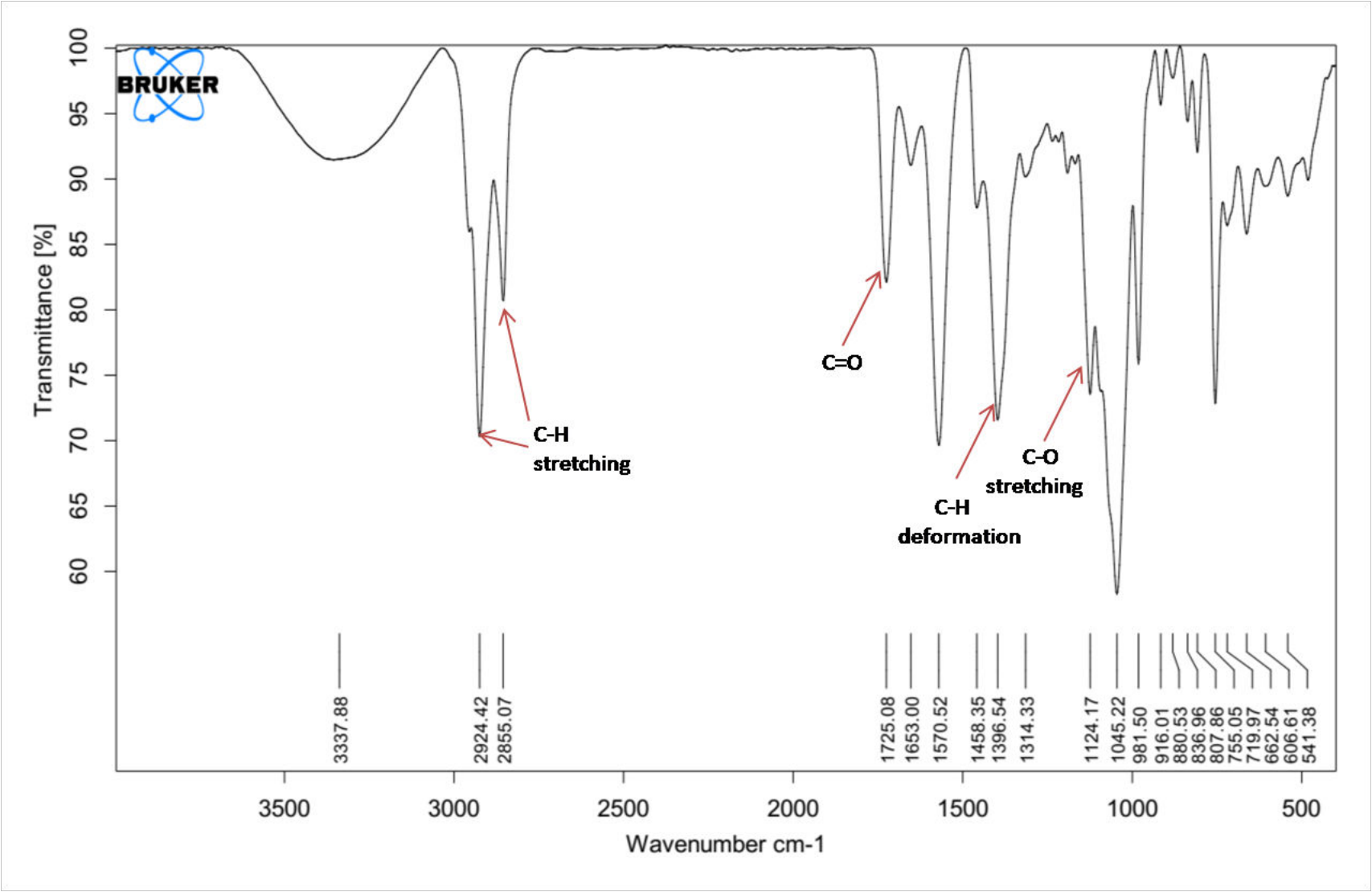

Supplement: FIGURE S1 — FTIR spectrum of commercial standard rhamnolipid. [file Image_1.TIFF]

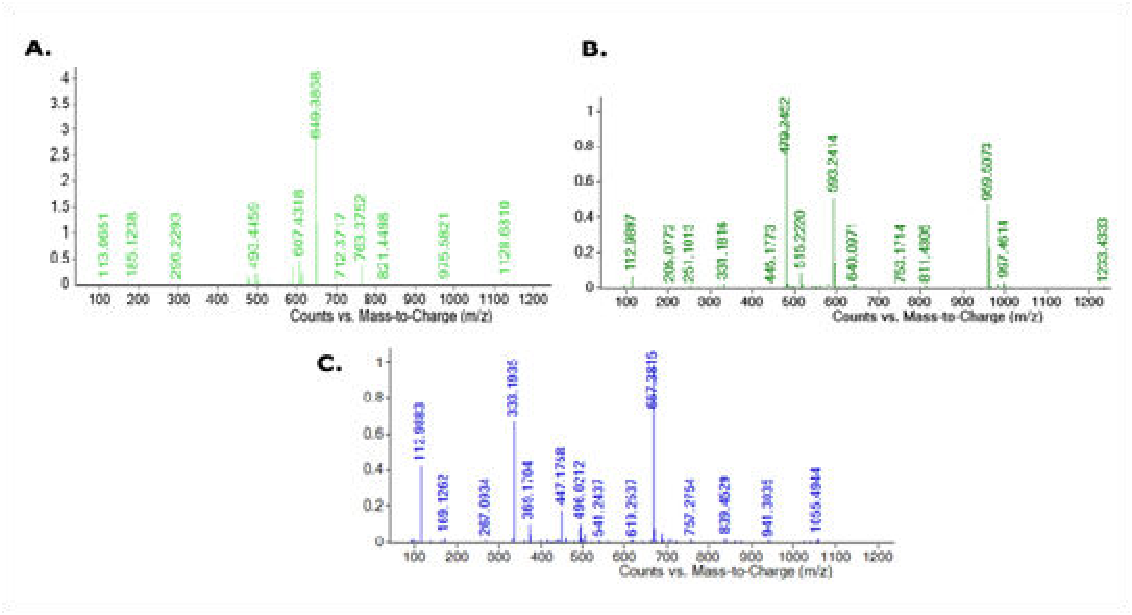

Supplement: FIGURE S2 — LC-MS spectrum of commercial Standard rhamnolipid. ESI-MS confirms the highest intensity Di-Rhamnolipid (A); Palmityl palmitate (B); Dipalmitin (C). [file Image_2.TIFF]
